# Retrospective cohort study of the association between socioeconomic deprivation and incidence of gestational diabetes and perinatal outcomes

**DOI:** 10.1186/s12889-023-17261-8

**Published:** 2024-01-15

**Authors:** Srirangan Jeyaparam, Rochan Agha-Jaffar, Edward Mullins, Ana-Catarina Pinho-Gomes, Kamlesh Khunti, Stephen Robinson

**Affiliations:** 1 Department of Metabolism, Digestion & Reproduction, Imperial College London, London, UK; 2 Department of Metabolic Medicine, Imperial College Healthcare NHS Trust, London, UK; 3 Department of Obstetrics & Gynaecology, Imperial College Healthcare NHS Trust, London, UK; 4 The George Institute for Global Health, London, UK; 5 University College London, London, UK; 6 University of Leicester, Leicester, UK

**Keywords:** Deprivation, Gestational diabetes mellitus, Perinatal outcomes, Birthweight centile

## Abstract

**Introduction:**

Socioeconomic disparities have been shown to correlate with perinatal mortality and the incidence of type 2 diabetes. Few studies have explored the relationship between deprivation and the incidence of gestational diabetes (GDM). We aimed to identify the relationship between deprivation and incidence of GDM, after adjusting for age, BMI, and ethnicity. We also examined for relationships between deprivation and perinatal outcomes.

**Methods:**

A retrospective cohort analysis of 23,490 pregnancies from a major National Health Service Trust in Northwest London was conducted. The 2019 English Indices of Multiple Deprivation was used to identify the deprivation rank and decile for each postcode. Birthweight centile was calculated from absolute birthweight after adjusting for ethnicity, maternal height, maternal weight, parity, sex and outcome (live birth/stillbirth). Logistic regression and Kendall’s Tau were used to identify relationships between variables.

**Results:**

After controlling for age, BMI & ethnicity, Index of Multiple Deprivation postcode decile was not associated with an increased risk of developing gestational diabetes. Each increase in decile of deprivation was associated with an increase in birthweight centile by 0.471 (*p* < 0.001). After adjusting for confounders, age was associated with a 7.1% increased GDM risk (OR: 1.076, *p* < 0.001); BMI increased risk by 5.81% (OR: 1.059, *p* < 0.001). There was no significant correlation between Index of Multiple Deprivation rank and perinatal outcomes.

**Discussion:**

Our analysis demonstrates that socioeconomic deprivation was not associated with incidence of GDM or adverse perinatal outcomes. Factors such as genetic predisposition and lifestyle habits may likely play a larger role in the development of GDM compared to socioeconomic deprivation alone.

**Supplementary Information:**

The online version contains supplementary material available at 10.1186/s12889-023-17261-8.

## Introduction

Gestational diabetes mellitus (GDM) is defined as glucose intolerance first recognised in pregnancy and leads to various degrees of hyperglycaemia during pregnancy [1]. Recent revisions to the definition have sought to recognise the increasing incidence of type 2 diabetes, which can present as diabetes during pregnancy [2].

The pathophysiology of gestational diabetes mellitus is thought to relate to an ineffective beta cell response to compensate for the increasing insulin resistance observed as gestation advances [3]. Factors that have been shown to increase the risk of GDM, overlap those associated with type 2 diabetes risk and include: obesity, advancing maternal age, a previous pregnancy complicated by GDM, polycystic ovary syndrome, non-white ethnicity, previous macrocosmic baby (> 4000 g) and a family history of type 2 diabetes mellitus (T2DM)) [3]. Hyperglycaemia during pregnancy is associated with adverse risk [4, 5], including increased neonatal adiposity [6], preeclampsia [7, 8], shoulder dystocia [9] and mechanical injuries during birth, resulting in fractures and nerve palsies [10]. Moreover, women with GDM have an increased lifetime risk of developing T2DM [11] and children of women exposed to in utero hyperglycaemia are predisposed to obesity [12] and diabetes [13] in later life.

The National Institute of Health and Clinical Excellence (NICE) suggests a two-stage approach in identifying women with gestational diabetes. In the first instance, women are screened for risk factors at their initial antenatal visit: those who have one or more risk factors are offered a 75 g 120-min oral glucose tolerance test. A fasting plasma glucose of 5.6 mmol/litre or above, and/or a 120-min plasma glucose level of 7.8 mmol/litre is considered diagnostic for GDM [14, 15].

As screening for GDM is essential to identify and manage the condition, healthcare professionals may need to consider the impact of socioeconomic status on GDM management. Disparities in social class have been shown to affect several health outcomes including life expectancy [16, 17], recovery from myocardial infarctions [18] and recovery from hip fractures [19]. In the context of type 2 diabetes mellitus, there is a clear association between socioeconomic deprivation and type 2 diabetes incidence [20]. People who live in areas with high levels of poverty and low educational attainment have been shown to have a higher risk of developing type 2 diabetes than those who live in more affluent areas [20]. Furthermore, deprivation has been shown to be an independent risk factor for the development of diabetes-related foot disease, (peripheral neuropathy, peripheral vascular disease, foot ulcers and lower limb amputation and gangrene) [21], as well as an effect magnifier for mortality in diabetes-related foot disease [22]. Additionally, a higher mortality is observed in people with T2DM living in deprived areas [23, 24].

The relationship between socioeconomic deprivation and the incidence of GDM and adverse perinatal outcomes can be influenced by mediating and moderating factors. Variables that act as intermediaries between the exposure (socioeconomic deprivation) and the outcomes (GDM and perinatal outcomes) are referred to as mediating factors. In this context, maternal diet is one example of a mediating factor. Women in low-income areas may have limited access to healthy food options and may consume diets high in calories but low in nutrients, which can increase their risk of GDM [25]. Additionally, maternal stress may be thought of as a mediating factor because it has been connected to an elevated incidence of GDM and unfavourable perinatal outcomes in women who reside in socioeconomically impoverished areas [26]. Another example of a mediating factor is maternal health behaviours, as women in socioeconomically disadvantaged areas may engage in behaviours such as smoking and alcohol consumption during pregnancy, which has been associated with an elevated risk of GDM and unfavourable perinatal outcomes [27].

Moderating factors are variables that affect the strength or direction of the relationship between the exposure (socioeconomic deprivation) and the outcome (GDM and perinatal outcomes). Maternal age is one example of a moderating factor. Older women are more likely to experience GDM [28], and the relationship between socioeconomic deprivation and GDM may be stronger in older women [29]. Another moderating factor is maternal BMI. Overweight and obese women have higher risks of GDM [30], and the relationship between socioeconomic deprivation and GDM may be stronger in women with higher BMIs [31]. Furthermore, maternal race/ethnicity can be viewed as a moderating factor as the relationship between socioeconomic deprivation and GDM may differ among different racial and ethnic groups [32].

In the context of pregnancy, deprivation has been shown to positively correlate with perinatal mortality [33]. The evidence relating to links between socioeconomic deprivation and the incidence of GDM is conflicting. One study utilised the Townsend Index in assessing material deprivation and found no correlation between material deprivation and the incidence of gestational diabetes in their cohort of 3933 women between 1996–1997 [34]. However, in an alternative study, which used median income of the maternal postcode area to measure deprivation, women of low-income backgrounds had a higher risk of developing gestational diabetes compared to women of high-income backgrounds [35]. These inconsistent findings imply that the relationship between socioeconomic deprivation and gestational diabetes is complex and may be influenced by both the methods used to assess for deprivation and the demographics of the population examined.

**Fig. 1:**
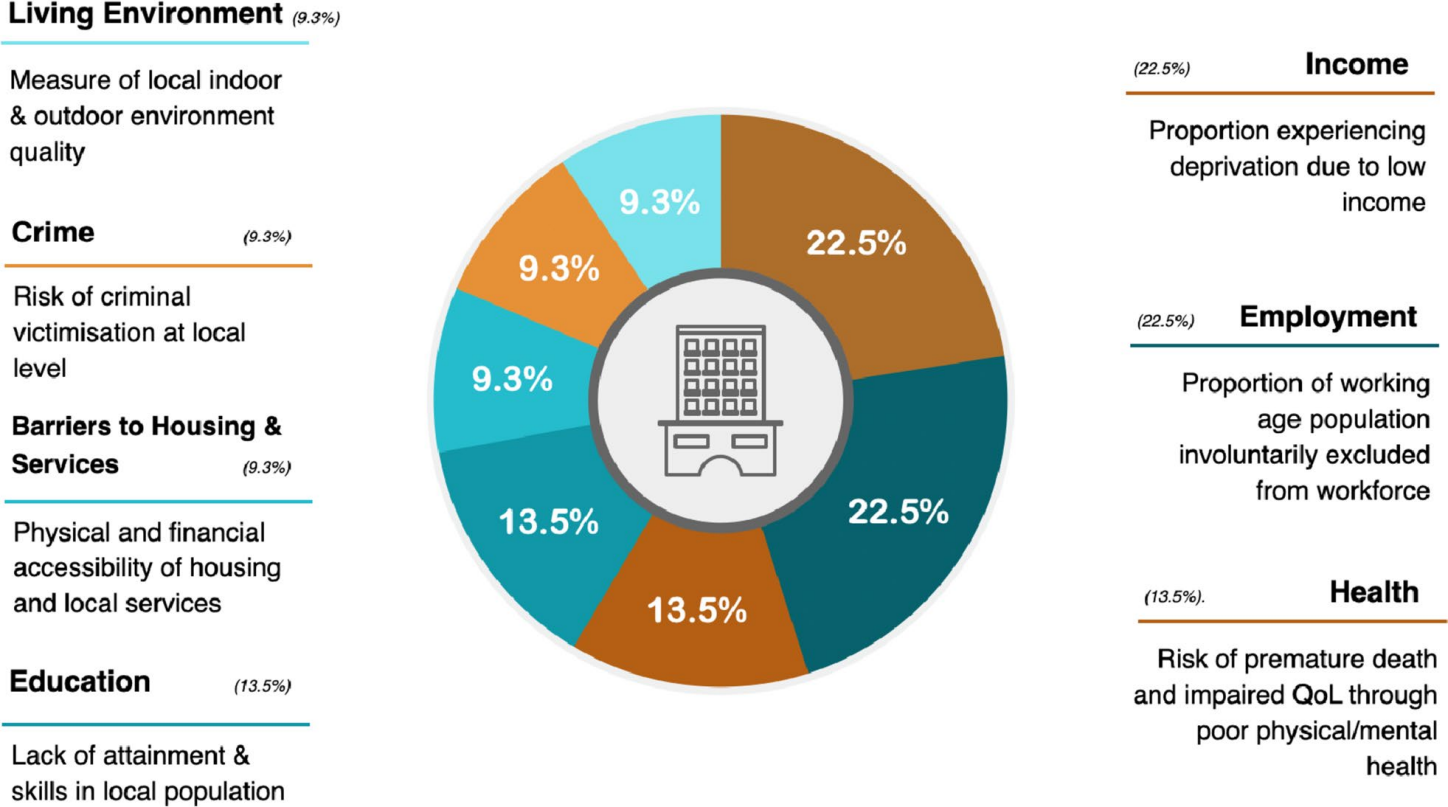
The domains and weightings which constitute the 2019 Index of Multiple Deprivation (IMD2019) Produced by Srirangan Jeyaparam, 2022. Additional information can be obtained from the U.K. Ministry of Housing, Communities and Local Government's website: https://assets.publishing.service.gov.uk/government/uploads/system/uploads/attachment_data/file/835115/IoD2019_Statistical_Release.pdf The U.K. Ministry of Housing, Communities and Local Government assigns Lower Layer Super Output Areas (LSOAs), which are small areas that constitute England and Wales, each with an average population of approximately 1500, a deprivation score using the IMD2019. This score is derived from 39 undisclosed indicators which are grouped into the above 7 domains of deprivation. These scores are then used to rank each LSOA nationally

Given the overlap in the risk factors for development of GDM and T2DM^3^, we hypothesised that socioeconomic deprivation would be associated with an increased incidence of GDM. We aimed to investigate the relationship between socioeconomic deprivation and incidence of GDM after adjusting for age, BMI, and ethnicity in a multi-ethnic cohort. To measure deprivation, we utilised the 2019 Index of Multiple Deprivation (IMD 2019), a validated deprivation index widely used in the United Kingdom to measure deprivation at the neighbourhood level [36] (Fig. 1). We also aimed to determine whether an association exists between deprivation and the following perinatal outcomes: birthweight centile (fetal birth weight adjusted for maternal and fetal demographics), still birth rate, admission to special care baby unit (SCBU) and proportion of neonates born preterm.

## Methods

### Study design and participants

We conducted a retrospective cohort analysis of the electronic patient health records (EPR) of women who registered their pregnancies at Imperial College Healthcare NHS Trust, London from April 2016 to Nov 2019.

**Fig. 2:**
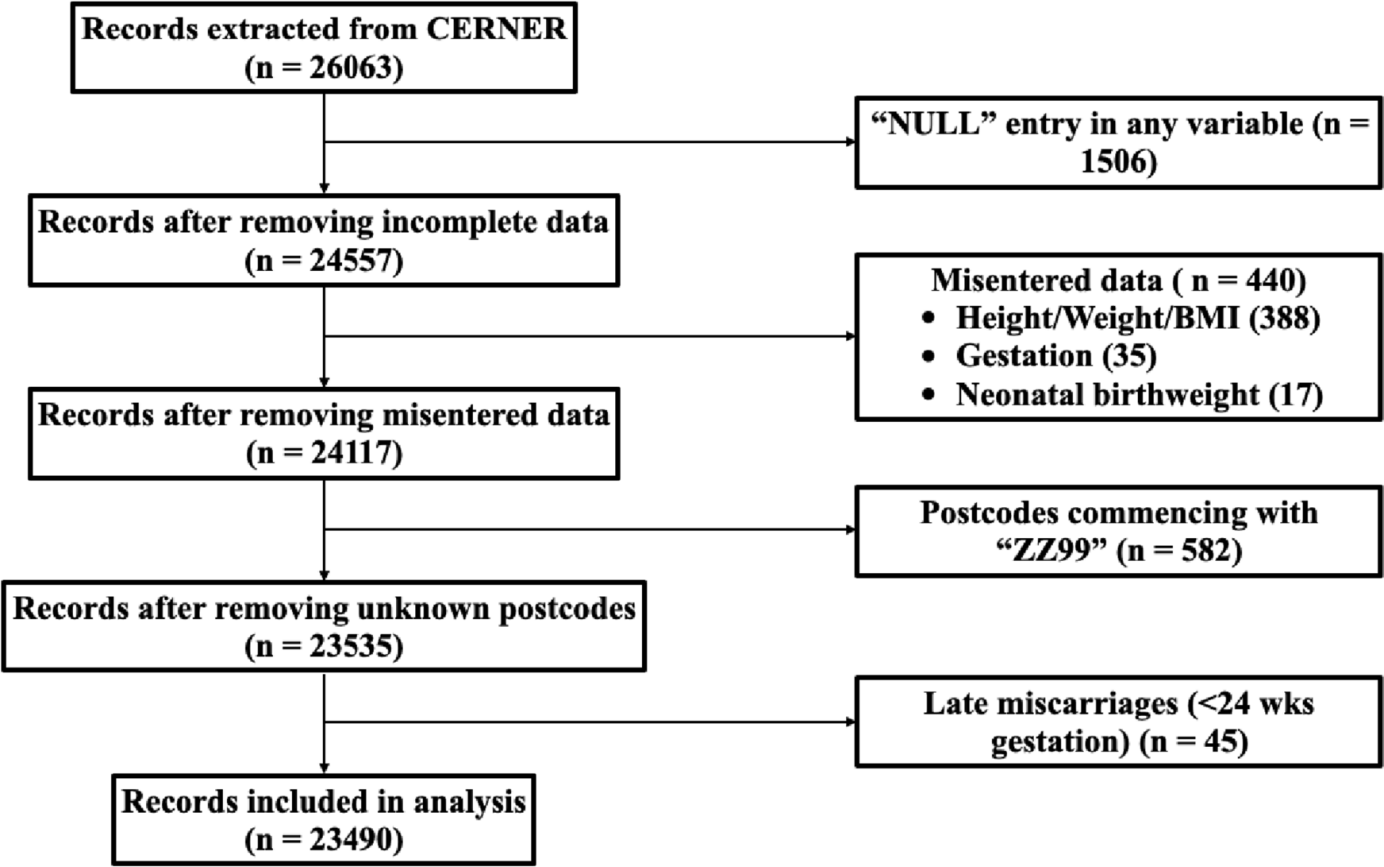
Flow chart showing inclusion process of the study 26063 records were extracted from the electronic health record system (Cerner). Records with any missing or misentered data in our required variables were removed from the dataset. Similarly, patients without a fixed address were also removed from the dataset. Late miscarriages (<24 weeks gestation) were also removed, leaving 23490 records for analysis

Initial search results yielded 26,063 patients, with the following variables available: postcode; age at start of pregnancy; maternal weight; maternal height; maternal BMI at booking; ethnicity (self-reported); parity; offer of glucose tolerance test; glucose tolerance test results (0 min and 120 min post 75 g glucose load); delivery modality; estimated total blood loss; gestational age; neonatal birthweight; SCBU admission; length of stay after delivery; fetal sex and stillbirth. Patients with missing values for one or more of the key variables were removed from the dataset prior to analysis and we did not attempt to impute missing data. Significantly outlying results were corrected where possible by re-examining original patient data: otherwise, datasets were removed. Inconsistencies in unit measurement were corrected. Patients with unknown or absent residential postcodes are conventionally marked with a postcode commencing with “ZZ99” [37, 38], hence for the purposes of this study, these patients were removed from the dataset. Late miscarriages (< 24 weeks gestation) were also removed due to challenges with accurately recording this outcome on the electronic system. 23,490 (90.13%) records were included in the final analysis (Fig. 2).

This project was registered with Imperial College Healthcare NHS Trust Audit and Governance Department. The dataset was anonymised prior to analysis.

The exposure of interest was socioeconomic deprivation, as defined by the IMD2019, and the outcomes measured were incidence of GDM and adverse perinatal outcomes. Predictors of interest included demographic factors such as age and BMI [39]. Lifestyle factors, such as physical activity and diet, and genetic factors [39] are also predictors of interest, however these were not included as we did not have data for these factors in our analysis. Other potential confounders were maternal smoking [40], alcohol use [41], and prenatal care utilisation [42]. Effect-modifying effects included the presence of obesity [43], hypertension [44], and other medical conditions that could impact the relationship between socioeconomic deprivation and GDM, and adverse perinatal outcomes. Due to unavailability of data in this dataset, we did not adjust for smoking, alcohol use, prenatal care utilisation, hypertension, or other medical conditions. We adjusted for age, BMI and ethnicity using our statistical analysis to accurately determine the presence of any associations.

### Procedures

#### Determining Fetal Birth weight centile

The birthweight centile for neonates in this study was calculated using the Perinatal Institute’s UK Bulk Centile Calculator (UK BCC) version 8.0.6.1, 2020. This tool uses maternal ethnicity, height, weight, parity, sex, gestation, absolute birthweight, and birth outcome to produce a customised birthweight centile for each neonate.

The UK BCC has 23 specified ethnic origins, however, the electronic medical record system, Cerner, in use at our centre codes for 16 different ethnicities. In most cases, the Cerner EHR recorded ethnicity had a corresponding UK BCC ethnic origin, but for certain groups, such as "White- Any Other White Background" or "Black- Any Other Black Background," there was no corresponding ethnic origin on the BCC tool. Therefore, means testing with sample data was used to determine the closest fit for these groups. Patients recorded in Cerner as "Asian—Other," "Mixed- any other mixed background," "Other- not stated," or "Other- any other ethnic group" were also assigned to the closest fitting ethnic origin on the BCC tool through means testing. Patients not fitting into any of the aforementioned categories were assumed to be of Middle Eastern ethnic origin: no separate category for this group existed on cerner and means testing was used to determine that this assumption was appropriate (see Appendix 1). Notably, all entries for ethnicity in Cerner were based on patients’ self-perceived ethnicity.

Postcodes were assigned their corresponding IMD rank and decile using the “Postcode Look Up Tool” provided online by the Ministry of Housing, Communities and Local Government [45]. The IMD rank and decile output for each postcode was added to the dataset.

### Statistical analysis

After testing for assumptions (see Appendix 2), we examined the associations between GDM and: IMD decile; age; BMI; and ethnicity using a multiple logistic regression model (see Appendix 3, for model details). Adjusted odds ratios (aORs) with 95% CIs for developing GDM were calculated for each of the following variables: IMD decile, age, BMI, and ethnicity, with IMD decile 10 (least deprived decile) and white- British as the references for IMD decile and ethnicity respectively. Multiple logistic regression was chosen to examine the associations between these predictor variables and the binary outcome of GDM, as this statistical test allows for the assessment of each predictor’s independent impact on GDM, while considering the influence of the other predictors.

Additionally, we employed a non-parametric test, Kendall’s tau-b, to examine the strength of relationships between IMD rank, gestational diabetes, age, BMI and ethnicity. A non-parametric test was appropriate as the variables did not have a normal distribution.

A simple ordinal logistic regression analysis was also performed to determine the relationship between IMD decile and birthweight centile. Given that birthweight centile already accounted for: maternal ethnicity, height, weight, parity, sex, gestation, absolute birthweight, and birth outcome, a simple ordinal logistic regression analysis allowed us to isolate the effect of IMD on birthweight centile while controlling for other relevant factors.

Lastly, a Kendall’s tau-b test was performed to assess the relationships between IMD rank, stillbirths, SCBU admissions and severe preterm births. Kendall’s Tau is well-suited for ordinal values, such as IMD rank, making it a logical choice for this aspect of the analysis also.

All analyses were performed using SPSS version 28.0.1.1.

## Results

 The mean (SD) age of our study population was 32.04 (± 5.53) years (Table 1). The median (IQR) early pregnancy BMI was 24.35 (6.45) kg/m^2^. In the study population: 2.96% had a BMI < 18.5 kg/m^2^), 52.35% had a BMI in the recommended range (BMI 18.5–24.9 kg/m^2^), 27.56% had an overweight BMI (BMI 25–29.9 kg/m^2^), and 17.13% had an obese BMI (BMI ≥ 30 kg/m^2^). 45.65% identified as White, 18.56% as Black, 21.22% as Asian, 3.42% as Mixed, and 11.16% as Other. Within the cohort, 56% were nulliparous and 2.61% were multiparous (parity >  = 4) (Table 2).

**Table 1:** Baseline maternal characteristics

*n* =	23,490
Mean (SD) Age at Start of Spell (years)	32.04 (5.53)
Mean (SD) Height (cm)	163.78 (6.87)
Mean (SD) Weight (kg)	68.24 (14.66)
Median (IQR) BMI	24.35 (6.45)
Mean (SD) Glucose Level (mmol/L) 0 min	4.35 (0.52)
Mean (SD) Glucose Level (mmol/L) 120 min	5.84 (1.54)
Mean (SD) Estimated Total Blood Loss (ml)	550 (422)
Mean (SD) Gestational Period (weeks)	38.97 (1.99)
Median (IQR) Index of Multiple Deprivation Rank	12,183 (11,361)
Median (IQR) Index of Multiple Deprivation Decile	4(3)

**Table 2:** Baseline maternal characteristics (continued)

	*n*	%
BMI^a^:		
Underweight (BMI < 18.5 kg/m^2^)	696	2.96
Healthy Weight (BMI 18.5 – 24.9 kg/m^2^)	12,297	52.35
Overweight (BMI 25.0 – 29.9 kg/m^2^)	6473	27.56
Obese (BMI ≥ 30 kg/m^2^)	4024	17.13
(Self-perceived) Ethnic Background:		
White	10,722	45.65
Black	4359	18.56
Asian	4984	21.22
Mixed	804	3.42
Other	2621	11.16
Parity:		
0	13,224	56.30
1–3	9652	41.09
≥ 4	614	2.61
Incidence of Gestational Diabetes	1854	7.89
Glucose Tolerance Test^b^		
Not Offered	5546	23.61
Offered and Accepted	17,844	75.96
Offered and Declined	100	0.43
Postcode Index of Multiple Deprivation Decile ^c^		
1	1199	5.1
2	3861	16.44
3	4019	17.11
4	3797	16.16
5	3068	13.06
6	2599	11.06
7	2015	8.58
8	1389	5.93
9	1053	4.48
10	490	2.09

^a^Weight measured at first booking

^b^75g 2-hour oral glucose tolerance test

^c^Decile a postcode’s deprivation rank falls into, according to IMD2019

The median Index of Multiple Deprivation rank was 12,183 (Table 1). The median Index of Multiple Deprivation decile was 4. 5.1% of the population resided in the top decile (decile 1) of least deprived areas and 2.09% in decile 10 (most deprived): the largest proportion of women (17.11%) resided in decile 3 (Table 2).

Following screening for GDM at the initial antenatal visit, 23.61% of the cohort did not require a 75 g 2-h oral glucose tolerance test. 75.96% were offered and subsequently accepted the glucose tolerance test: 0.43% declined the test. The incidence of index gestational diabetes in this study population was 7.89%. Mean (SD) fasting plasma glucose level measured 4.35 (± 0.52) mmol/L and the mean (SD) 2-h glucose level 5.84 (± 1.54) mmol/L (Tables 1 and 2).

In terms of materno-fetal outcomes, mean (SD) estimated blood loss measured 550 (± 422) ml (Table 1). 46.81% were estimated to have mild postpartum haemorrhage (500-1000 mL), and 6.93% were estimated to have major postpartum haemorrhage (> 100 mL). The delivery modalities were as follows: 53.96% had a spontaneous vaginal delivery, 14.71% assisted vaginal delivery. 17.08% of women required an emergency caesarean section and 14.25% had an elective caesarean section (Table 3).

**Table 3:** Maternal delivery outcomes

	*n*	%
Mode of Delivery:		
Spontaneous Vaginal Delivery	12,676	53.96
Emergency Caesarean	4012	17.08
Assisted Vaginal Delivery	3455	14.71
Elective Caesarean	3347	14.25
Estimated Total Blood Loss:		
Mild postpartum Haemorrhage (500 mL-1000 mL)	10,996	46.81
Major Postpartum Haemorrhage (> 1000 mL)	1627	6.93

The mean (SD) absolute birthweight of the 23,490 neonates was 3275.02 (± 573.54) grams (Table 4). The median gestational age was 39 weeks. 93.05% neonates were born at term (≥ 37 weeks), 5.79% were born moderate-late preterm (32–37 weeks), and 1.17% were born very preterm (< 32 weeks) (Table 5).

**Table 4:** Baseline neonatal characteristics

*n* =	23,490
Mean (SD) Absolute Birthweight (grams)	3275.02 (573.54)
Median (IQR) Birthweight Centile	48.1 (52.3)
Median (IQR) Gestational Age (weeks)	39 (2)
Stillbirths (rate per 1000)	5.79

The median adjusted birthweight centile was 48.1: 8.01% of neonates were born macrosomic (> = 4000 g) (Table 5). 11.73% of neonates were born large for gestational age (≥ 90^th^ centile) and 11.52% were born small for gestational age (< 10^th^ centile). The calculated stillbirth rate was 5.79 per 1000: 0.58% of neonates within the cohort were stillborn. Shoulder dystocia complicated delivery in 1.16% of neonates: 5.51% were admitted to the special care baby unit (Table 4).

**Table 5:** Baseline neonatal characteristics (continued)

	*n*	%
Gestational Age:		
Normal	21,857	93.05
Preterm (< 37 weeks)	1259	5.79
Severely Preterm (< 32 weeks)	274	1.17
Sex:		
Male	12,080	51.43
Female	11,410	48.57
Birthweight:		
Absolute birthweight ≥ 4 kg	1882	8.01
Small for Gestational Age (< 10^th^ Centile^a^)	2705	11.52
Appropriate for Gestational Age	18,029	76.75
Large for Gestational Age (≥ 90^th^ Centile^a^)	2756	11.73
Live birth	23,354	99.42
Stillborn	136	0.58
Shoulder Dystocia	273	1.16
Admitted to SCBU^b^	1295	5.51

^a^Customised neonatal birthweight centile adjusted for maternal height, maternal weight, maternal ethnicity, maternal parity, neonatal sex, gestational period, absolute birthweight, and birth outcome (live birth/stillbirth)

^b^Special Care Baby Unit

After adjusting for age, BMI, and ethnicity, there was no significant association between IMD decile and odds of developing gestational diabetes. After adjusting for BMI, IMD decile, and ethnicity, age was associated with an increase in the odds of developing gestational diabetes (aOR 1.076; 95% CI 1.066–1.086; *P* < 0.001). BMI showed an association with increased odds of gestational diabetes (aOR 1.059; 95% CI 1.050–1.069; *P* < 0.001).

**Fig. 3:**
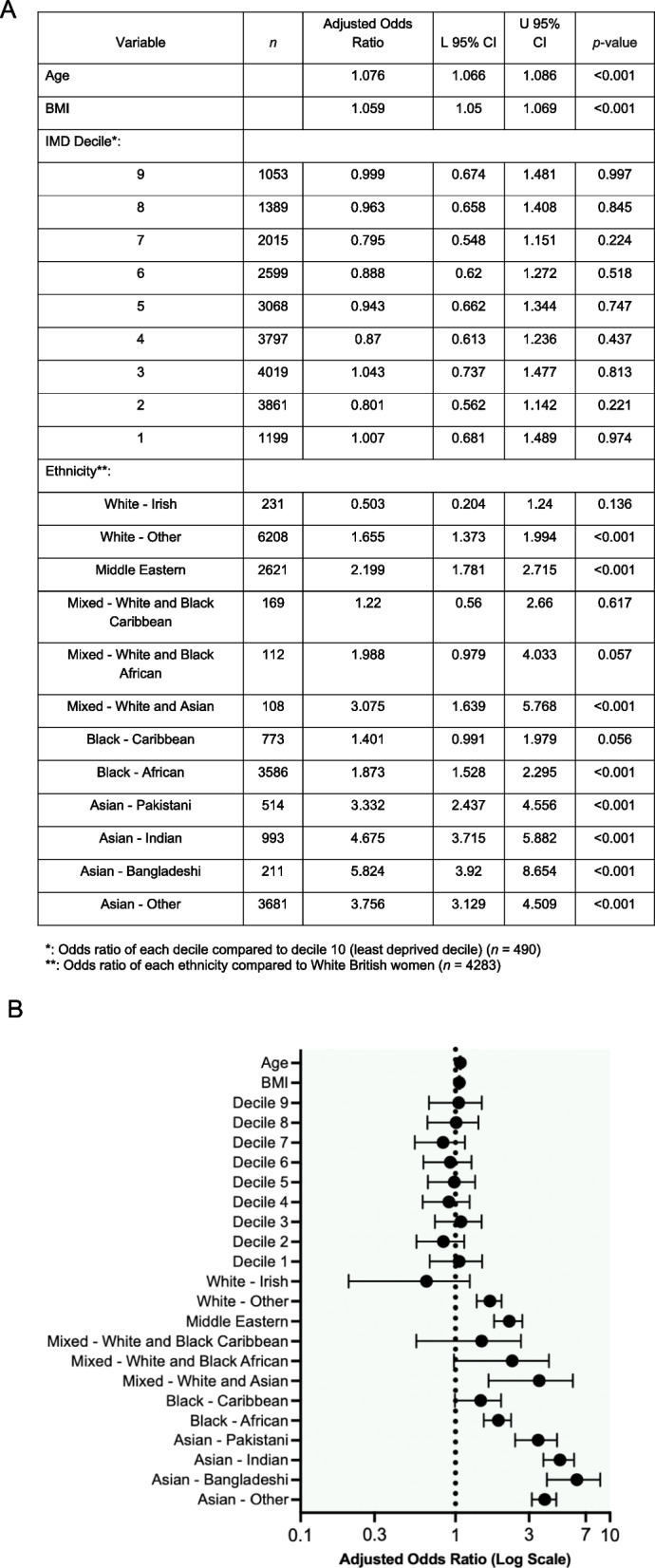
**A** Table and **B** Forest plot showing adjusted odds ratios from multiple logistic regression model Multiple logistic regression analysis was conducted with the above variables. There was no significant difference in odds between any of the postcode deprivation deciles (as determined by IMD2019), and their odds of developing GDM, compared to decile 10 (least deprived decile). Age and BMI independently increased odds of developing GDM. Similarly, only women from the following ethnic groups had significantly higher odds of developing GDM compared to White British women: White- Other, Middle Eastern, Mixed- White and Asian, Black- African, Asian- Pakistani, Asian- Indian, Asian- Bangladeshi and Asian- Other

Ethnicity significantly impacted the odds of developing gestational diabetes. After controlling for age, BMI, and IMD decile, compared to White British women, the odds of developing gestational diabetes were significantly higher for White-Other (aOR 1.655; 95% CI 1.373–1.994; *P* < 0.001), Middle Eastern (aOR 2.199; 95% CI 1.781–2.715; *P* < 0.001), Mixed- White and Asian (aOR 3.075; 95% CI 1.639–5.768: *P* < 0.001), Black- African (aOR 1.873; 95% CI 1.528- 2.295; *P* < 0.001), Asian- Pakistani (aOR 3.332; 95% CI 2.437–4.556; *P* < 0.001), Asian- Indian (aOR 4.675; 95% CI 3.715–5.882; *P* < 0.001), Asian-Bangladeshi (aOR 5.824; 95% CI 3.920–8.654; *P* < 0.001), and Asian- Other (aOR 3.756; 95% CI 3.129–4.509; *P* < 0.001) women. There was no significant difference in the odds of developing gestational diabetes for White- Irish, Mixed- White and Black Caribbean, Mixed- White and Black African, and Black- Caribbean women compared to White British women (Fig. 3).

Kendall’s Tau showed no significant correlation between IMD rank and gestational diabetes incidence (Kendall’s Tau = 0.000; *p*-value (2-tailed) = 0.932) (Table 6).

**Table 6:** Kendall’s Tau correlation analysis between IMD Rank, Gestational Diabetes Incidence, Age, BMI and Ethnicity

		IMD Rank	Gestational Diabetes	Age	BMI	Ethnicity
IMD Rank	Correlation coefficient	1.000	0.000	0.116^a^	-0.098^a^	-0.092^a^
	Sig. (2-tailed)	-	0.932	< 0.001	< 0.001	< 0.001
Gestational Diabetes	Correlation coefficient	0.000	1.000	0.085^a^	0.079^a^	0.101^a^
	Sig. (2-tailed)	0.932	-	< 0.001	< 0.001	< 0.001
Age	Correlation coefficient	0.116^a^	0.085^a^	1.000	0.018^a^	-0.059^a^
	Sig. (2-tailed)	< 0.001	< 0.001	-	< 0.001	< 0.001
BMI	Correlation coefficient	-0.098^a^	0.079^a^	0.018^a^	1.000	0.093^a^
	Sig. (2-tailed)	< 0.001	< 0.001	< 0.001	-	< 0.001
Ethnicity	Correlation coefficient	-0.092^a^	0.101^a^	-0.059^a^	0.93^a^	1.000
	Sig. (2-tailed)	< 0.001	< 0.001	< 0.001	< 0.001	-

^a^Correlation is significant at the 0.01 level (2-tailed)

Simple ordinal logistic regression analysis between IMD decile and birthweight centile demonstrated an increase in birth weight centile by 0.471 for every increase in decile (unstandardised beta coefficient = 0.471; 95% CI 0.303–0.639; *p*-value (2-tailed) < 0.001) (Table 7).

**Table 7:** Ordinal Logistic regression analysis between IMD Decile and birthweight centile

	Birthweight Centile			
Independent Variable	Unstandardised coefficient	Std. Error	*p*-Value	95% CI
IMD Decile	0.471	0.086	< 0.001	[0.303 – 0.639]

There was no significant correlation between: IMD rank and stillbirths (Kendall’s Tau b = 0.004; *p*-value (2-tailed) = 0.410), SCBU admissions (Kendall’s Tau b = -0.009; *p*-value (2-tailed) = 0.085), and severe preterm births (Kendall’s Tau b = -0.005; *p*-value (2-tailed) = 0.366) (Table 8).

**Table 8:** Kendall’s Tau correlation analysis between IMD Rank, Stillbirths, SCBU Admissions and Severely Preterm births

		IMD Rank	Stillbirth	SCBU Admissions	Severe Prematurity
IMD Rank	Correlation coefficient	1.000	0.004	-0.009	-0.005
	Sig. (2-tailed)	-	0.410	0.085	0.366
Stillbirth	Correlation coefficient	0.004	1.000	0.004	0.269^a^
	Sig. (2-tailed)	0.410	-	0.571	0.000
SCBU Admissions	Correlation coefficient	-0.009	-0.009	1.000	0.351^a^
	Sig. (2-tailed)	0.085	0.085	-	0.000
Severe Prematurity	Correlation coefficient	-0.005	0.269^a^	0.351^a^	1.000
	Sig. (2-tailed)	0.366	0.000	0.000	-

^a^Correlation is significant at the 0.01 level (2-tailed)

## Discussion

Associations between socioeconomic deprivation and poor health outcomes are well documented. While a clear relationship exists between deprivation and type 2 diabetes (T2DM) and its related complications, the evidence relating to the impact of deprivation on gestational diabetes is conflicting.

The overall incidence of index gestational diabetes (GDM) in our multi-ethnic cohort of 23,490 women was 7.89%. After controlling for age, BMI, and ethnicity, deprivation as determined by the IMD2019 decile was not associated with GDM risk. Similarly, we found no association between deprivation and adverse neonatal outcomes.

Our hypothesis that GDM incidence would be adversely affected by socioeconomic deprivation was based on the overlap between the pathophysiology of GDM and T2DM as well as the risk factors that contribute to their development. There are limitations in our study, which could account for the negative finding. In the first instance, while our study was able to include predictors of interest such as age and early pregnancy body mass index, lifestyle factors such as physical activity and diet were not included. Genetic factors could not be adjusted for and effect-modifying factors such as hypertension and other medical comorbidities including polycystic ovary syndrome that contribute to GDM risk were not adjusted for in the analysis. Furthermore, environmental aspects that could contribute to risk e.g. pollutants were not examined.

Our analysis showed a weak, but statistically significant negative correlation between IMD rank and BMI, suggesting those residing in more socioeconomically deprived areas were leaner, which may additionally explain why GDM did not appear to be more prevalent in these areas.

In congruence with other studies [46, 47], age and early pregnancy body mass index were independently associated with an increased risk of GDM, which was also supported by the Kendall’s Tau correlation analysis.

Our study additionally highlights the increased risk of developing gestational diabetes in various ethnic groups. Similar to other studies [48, 49], our results demonstrated that Asian women in particular had the highest risk of developing GDM. This group (including women of mixed Asian ethnicity) were more than three times as likely to develop GDM compared to White British women, even after controlling for age, IMD decile and BMI. South Asian populations have higher incidences of T2DM [50, 51], insulin resistance [52], and impaired fasting glycemia [53, 54] which likely explains the increased incidence of GDM in these ethnic groups.

Our analysis demonstrated an association between deprivation and birth weight centile, with birth weight centile being shown to increase as deprivation increased, irrespective of whether an individual was diagnosed with GDM. This could in part relate to the pitfalls of testing for gestational diabetes in pregnancy and perhaps highlights the fact that complications of reduced insulin sensitivity e.g. fetal macrosomia persist in “at risk” women even in the absence of maternal hyperglycaemia. Importantly though, there was no association between deprivation and perinatal morbidity and mortality indicating that this increase in birth weight centile did not have an associated adverse effect on the neonate in the immediate postpartum period.

A key strength of our study is that our method of measuring deprivation (the IMD 2019) is less heterogeneous within a measured area than other methods, such as the Carstairs Index, because it uses smaller population sizes (1,500 on average [36]) to measure deprivation. It is worth noting that the 2019 IMD is a relative measure of deprivation [36] and ranks LSOAs nationally. It is possible there is a “threshold level” of deprivation below which there may be a significant association between socioeconomic deprivation and the incidence of GDM, which may be seen in other, less developed countries. If such a threshold exists, our study sample may have registered above this threshold, which could explain the absence of a relationship between deprivation and GDM in our study.

There are further limitations in our study that should be considered. We made one key assumption for the neonatal birthweight centile calculator and that was that those recorded on the electronic patient record as “Other- any other ethnic group” and “Other- not stated” were of Middle Eastern ethnicity. This was based on an analysis of a sample size, which indicated a large proportion of our women are of Arab/ Middle Eastern ethnicity: no coding exists for this sub-set on Cerner. Consequently, if there were patients that belonged to other, smaller ethnic groups such as indigenous peoples, they may have been misclassified in the neonatal calculator. Additionally, as the calculator had no input for paternal factors, neonatal ethnicity was assumed to be the same as maternal ethnicity, which may be misrepresentative of neonates born to mixed-ethnicity couples. Finally, ethnicity was self-reported.

Further limitations comprise the exclusion of women of no fixed abode (“ZZ99” postcode, *n* = 582). These women were excluded on the basis that they could not be assigned a deprivation rank or decile: however, they are likely to represent a socioeconomically deprived group, therefore exclusion of this group may impact the generalisability of our findings. Furthermore, complex social circumstances such as asylum seeker status and those exposed to domestic violence could not be coded for and may have had a bearing on the results. Limitations additionally exist in the coding for neonatal outcomes. While special care baby unit admissions, still births and preterm birth rates could be accurately determined, the incidence of shoulder dystocia is likely to be under-represented: coding for this relies on healthcare professionals inputting data accurately retrospectively.

In conclusion, we have shown that, after adjusting for age, BMI and ethnicity, there was no significant association between socioeconomic deprivation and the incidence of developing gestational diabetes. Furthermore, we have shown the independent effects of age, BMI, and ethnicity on the development of gestational diabetes. Lastly, we have shown that there was no correlation between socio-economic deprivation and adverse neonatal outcomes.

Studying the associations between socioeconomic deprivation and the incidence of gestational diabetes mellitus and adverse perinatal outcomes is important as it provides insights into the social determinants of health and helps inform interventions aimed at reducing disparities in maternal and child health outcomes. Understanding these relationships can also inform healthcare policies and improve clinical practice, leading to better health outcomes for women and their children. By examining the impact of socioeconomic status on the incidence of GDM and perinatal outcomes, researchers can identify populations at higher risk for these conditions and develop targeted interventions to improve outcomes. The results of these studies also inform healthcare providers and policymakers on the development and implementation of effective strategies to reduce health disparities and improve outcomes for all individuals and communities.

### Supplementary Information

**Additional file 1: Appendix 1. **Breakdown of Cerner electronic health record ethnicity to neonatal birthweight centile calculator ethnic origin input. **Appendix 2. **Assumptions testing for multiple logistic regression model. **Figure S1.** Assumptions test for multicollinearity (Tolerance & Variance Inflation Factor). **Figure S2.** Assumptions test for outliers (ROUT, Q=1%). **Appendix 3. **Multiple regression model details.

## Data Availability

The datasets generated and/or analysed during the current study are available in the figshare repository, https://doi.org/10.6084/m9.figshare.21806472. Additional analyses data is available from the corresponding author on reasonable request.
