# Supplementary material for: Retrospective cohort study of the association between socioeconomic deprivation and incidence of gestational diabetes and perinatal outcomes
Source: BMC Public Health. 2024 Jan 15;24:184. doi: 10.1186/s12889-023-17261-8 (PMC10790393; doi:10.1186/s12889-023-17261-8)

**Appendices:**

**Appendix 1. Breakdown of Cerner electronic health record ethnicity to neonatal birthweight centile calculator ethnic origin input**

**
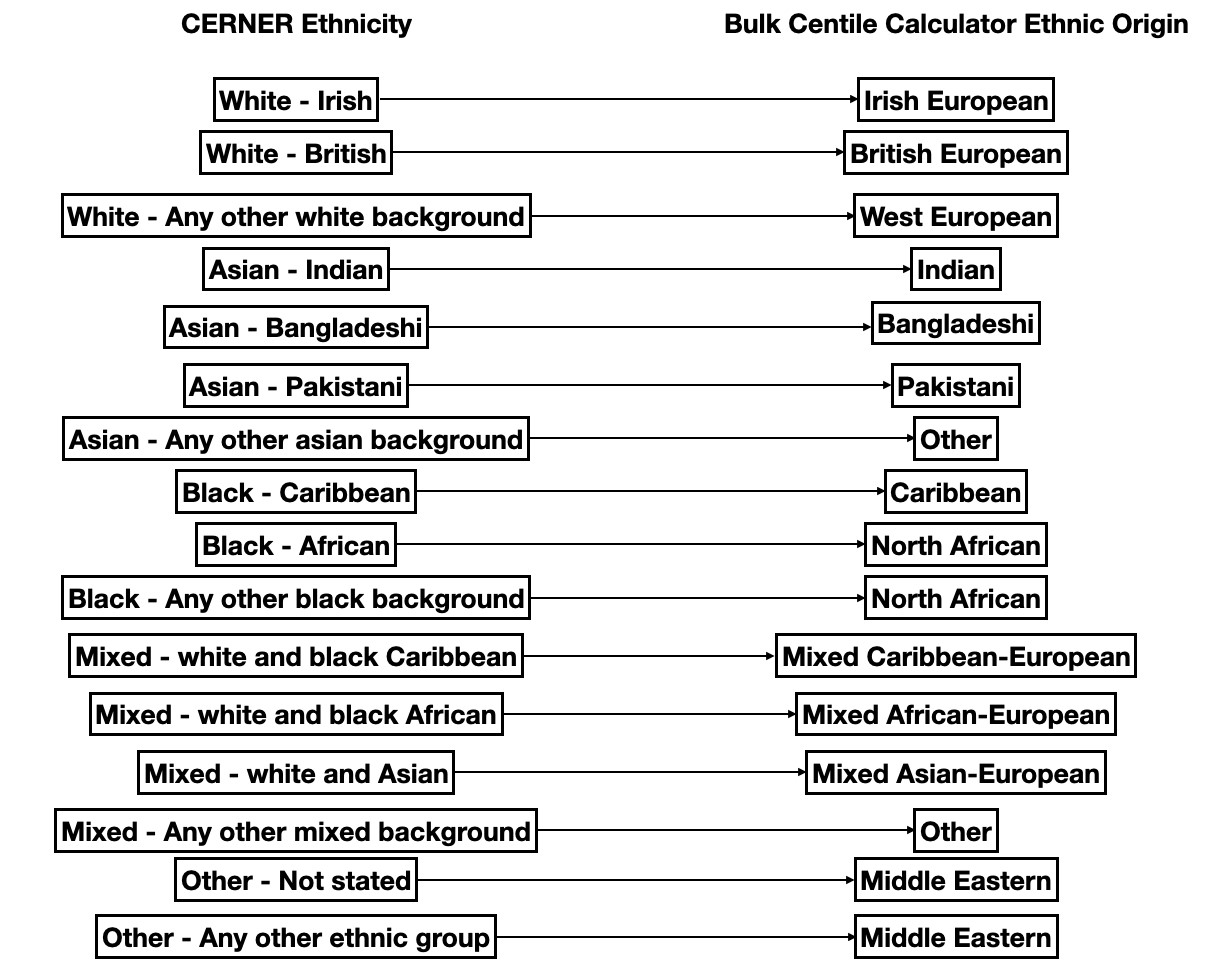
**

**Appendix 2. Assumptions testing for multiple logistic regression model**

Figure S1. Assumptions test for multicollinearity (Tolerance & Variance Inflation Factor)

|  | Collinearity Statistics | |
| --- | --- | --- |
| Variable | Tolerance | VIF |
| Age | 0.967 | 1.034 |
| Ethnicity | 0.997 | 1.003 |
| BMI | 0.972 | 1.029 |
| Index of Multiple Deprivation Decile | 0.947 | 1.056 |

Figure S2. Assumptions test for outliers (ROUT, Q=1%)


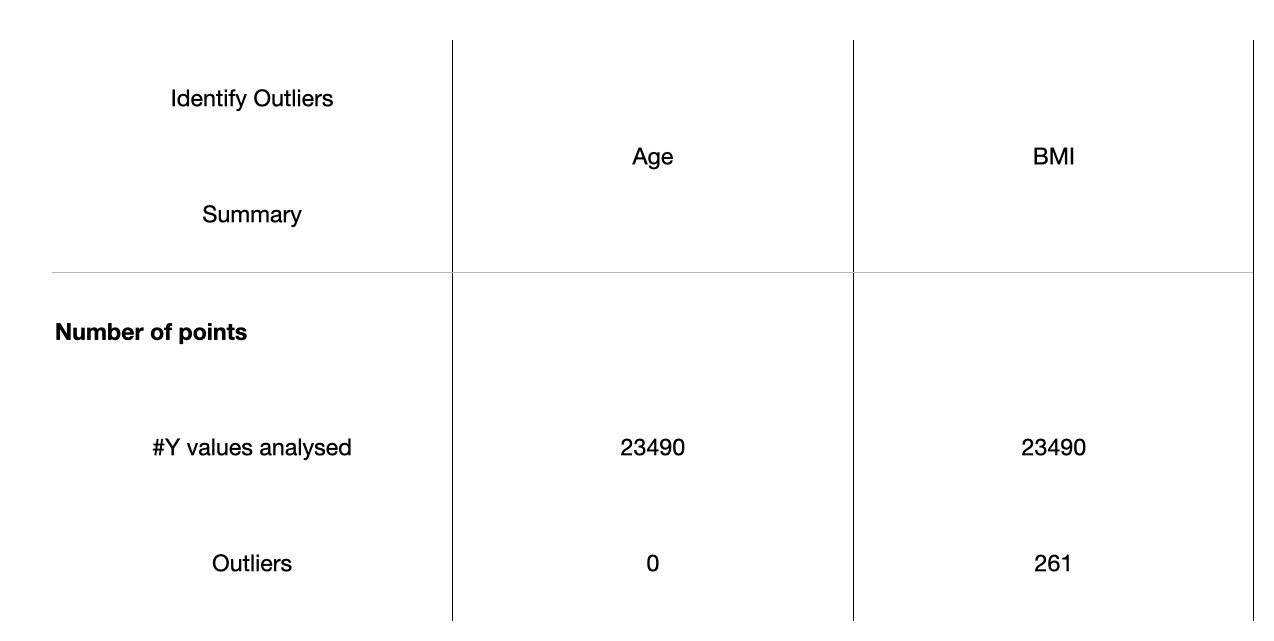


**Appendix 3. Multiple regression model details**


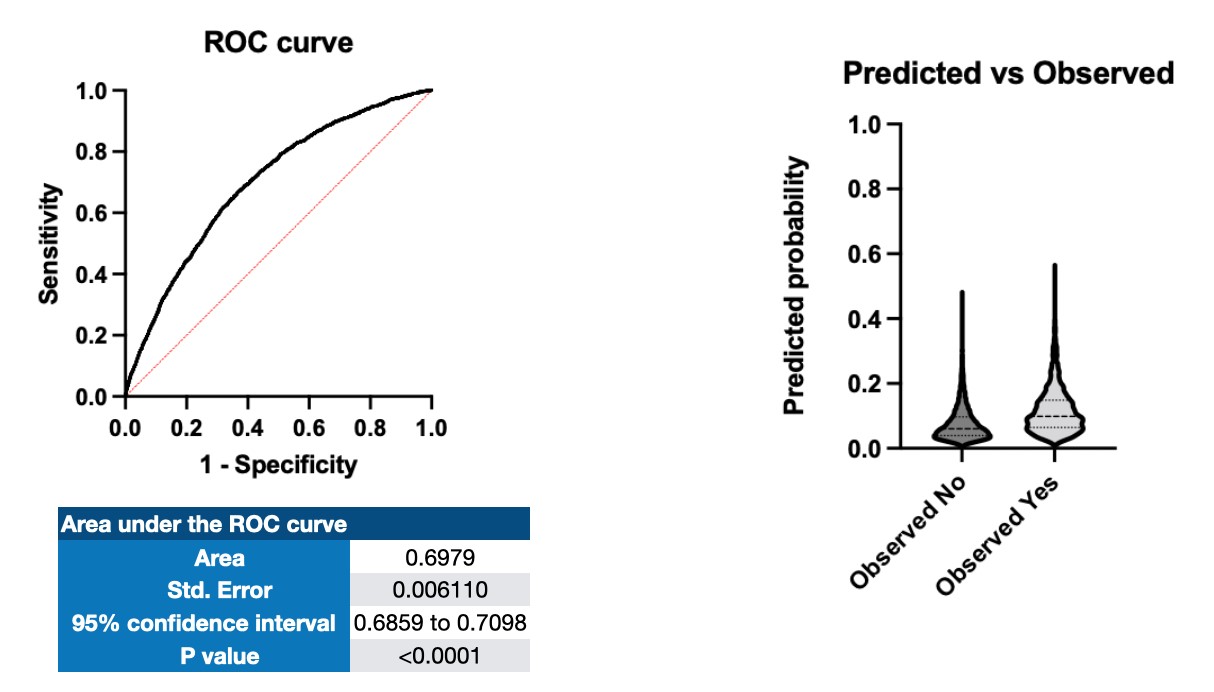

Supplement: Supplementary file 1 — Additional file 1: Appendix 1. Breakdown of Cerner electronic health record ethnicity to neonatal birthweight centile calculator ethnic origin input. Appendix 2. Assumptions testing for multiple logistic regression model. Figure S1. Assumptions test for multicollinearity (Tolerance & Variance Inflation Factor). Figure S2. Assumptions test for outliers (ROUT, Q=1%). Appendix 3. Multiple regression model details. [file 12889_2023_17261_MOESM1_ESM.docx]
